# Sensory sacrifices when we mass-produce mass produce

**DOI:** 10.1038/hortres.2016.32

**Published:** 2016-07-13

**Authors:** Kevin M Folta, Harry J Klee

**Affiliations:** 1 Horticultural Sciences Department, Plant Innovation Center and The Graduate Program for Plant Molecular and Cellular Biology, University of Florida, Gainesville, FL 32606, USA

## Abstract

Plant breeders have been extremely successful at driving genetic improvements in crops. However, ‘improvements’ are truly a question of perspective. Over the last one-hundred years most plant genetic innovations have been driven by industry demand. Larger fruits, heavier yields, uniformity, increased resistance to disease and better shipping quality are just a few of the traits that have ensured profits on the farm and affordable food for consumers. However, these milestones have come at the expense of sensory qualities, which have been sacrificed in exchange for practical production objectives. With a base of industry-sufficient genetics, today’s breeders can now turn to the consumer for guidance in defining critical desires. New approaches to plant breeding start with the analysis of consumer preferences, and then merge them with modern genomics and analytical chemistry tools. The result is the next generation of crops that meet supply chain demands while presenting improvements in flavor, nutrition, color, aroma and texture. This review analyzes the approach of consumer-assisted selection as it has been applied to tomato and strawberry, two complementary annual crops that have been intensively bred to meet industry expectations. Current breeding efforts start with the consumer, with the objective of reclaiming lost sensory qualities.

Henry Ford will long be remembered as the inventor who brought a transformational product to the masses. The Ford Motor Company’s assembly line strategy was a breakthrough in manufacturing, creating new vehicles in hours instead of days and fueling new avenues in consumer desire. Greater throughput drove lower costs for automobiles, leading to wider availability for willing consumers. But to keep production costs in check, Ford had to impose certain sacrifices in areas that constrained consumer preference while not affecting core product qualities. In 1909, Ford announced that there would be one car, the Model T, and that it would be available in only one standard body type. He also remarked, ‘Any customer can have a car painted any color that he wants so long as it is black’.^1^

Ford’s statement frames an important dilemma. The efficient, low-cost production of in-demand items often comes with trade-offs. Personal preferences and sensory attributes might be sacrificed to keep a product inexpensive and widely available. The Model T is a metaphor for today’s fresh fruit and vegetable market. An unprecedented bounty of produce items have rolled-off of plant breeding’s assembly line, constructed of the best genetics that deliver an inexpensive, accessible and typically satisfactory product. We have mastered the art of how to mass-produce mass produce.

Today the demands for fresh produce arise from increasingly curious and discerning consumers. There is a growing interest in food, farming and how fruits and vegetables can be an important part of a healthy diet. However, with all of the excitement around fruits and vegetables, consumers believe that there is plenty of room for improvement.^2^ Although consumers are generally pleased with price, availability and safety, the core criticisms relate to flavors and aromas. Consumers consistently note that produce lacks favorable sensory qualities, and that is no surprise. Over the last 50 years, plant breeders have made tremendous progress satisfying objectives of an ambitious supply chain that demands large, perfect, abundant products, with year-round availability. This strict focus on production metrics coincides with erosion of the genetic and biochemical complexity essential to fruit sensory quality. Today’s elite varieties have been bred to produce high yielding, disease-free plants, producing easily harvested products that resist decay and ship smoothly within and across nations. While production has been priority one, flavor and nutrition have suffered from benign neglect. The irony has been described as, ‘The modern supermarket tomato became damaged goods as part of the attempt to keep it from becoming damaged goods’.^3^ In the past, if sensory quality was acceptable in a plant that yielded perfect, big and long-lasting fruits—the breeding effort was remarkably successful. Consumers asked for cheap, always available produce and the breeders gave them what they wanted; consumers asked for the Model T, and scientists and farmers provided the Model T. Only today, consumers are thinking about tricked out Bel-Airs with tail-fins and Mustang convertibles.

Breeders most certainly did not set out to produce fruits and vegetables with poor flavor. Breeding for sensory quality was deprioritized because of its complexity. Hundreds, if not thousands, of chemical compounds contribute to the flavor and aroma, and interlace with sensations triggered by acids and sugars.^4–6^ Humans exhibit great variation in their sensory perception.^7^ Flavor breeding requires a combination of expensive analytical tools as well as a deep understanding of human sensory psychology and access to consumer evaluation panels. The presence of specific compounds that shape flavor and aroma are informative, as they may relate to the nutrient content of the food item.^8^ Overlay physiological factors that disrupt chemical sensing or perception, age, learned preferences, social influences, texture and visual cues, and it is no wonder that flavor and aroma are such daunting breeding targets.^9^ The goal now is not solely to create good products that satisfy the consumer and the industry. Instead the target is to produce fruits and vegetables that consumers actively seek, while maintaining industry-mandated qualities.

The idea of putting the consumer first is shockingly novel, but it is an essential first step in growing demand for fruits and vegetables rather than merely maintaining existing markets (Figure 1). Bringing the consumer into breeding strategies requires an interdisciplinary approach with a long-term vision. Measuring what people actually like requires specific knowledge of the way that ‘liking’ is measured and related to chemical content of the product. The classical concept of measuring a volatile compound’s relevance to flavor by integrating concentration with presumptive human sensory thresholds (described as ‘odor units’) has some important limitations (reviewed in Bartoshuk and Klee, 2013).^10^ The ranking system is too simple; every consumer’s sensory receptor collection is different. Integration of sensory inputs in the brain with memories, biases and experiences shapes consumer liking. Relating chemical composition with a liking score across a population requires sophisticated measurement tools. Hedonic scaling tools have led to breakthroughs in linking the chemistry of flavor to breeding efforts in tomato,^11^ strawberry^12^ and melon.^13^ These consumer-based approaches provide a means to prioritize flavor compound targets for breeding.

This review examines consumer evaluations and the molecular basis for flavor in tomato and strawberry, two high-value crops that are recognized as having great opportunity for sensory improvement. These two crops have much in common regarding research and breeding for fruit quality (Figure 2). They are both intensively bred annuals, meaning that in the absence of positive selection for flavor quality, there is a greater risk for flavor deterioration in the race to produce larger fruits and higher-yielding varieties. Both systems are readily transformable for *in planta* validation of gene function.^14,15^ Genome sequences of the species and of close relatives is available,^16,17^ and there is a rich diversity of transcriptome resources in both systems. Strawberry and tomato are logically complementary, as both high-value fruits are remarkably different in botanical development. Strawberry is technically a vegetable, and tomato is technically a berry fruit. The mechanisms of sensory compound production and their roles in flavor and aroma may also contrast in these two different products. Here we discuss how researchers have unraveled complex questions in flavor and aroma by making consumer and sensory traits the priority in the breeding process. The work outlined has led to the use of new biochemical-genetic-genomic tools to funnel sensory-relevant genes into advanced selections, using molecular breeding to hasten the process.

## Tomatoes

Tomato flavor is defined by a complex blend of key volatiles that intertwine with sugars and acids.^5^ The interaction between them is played out in concert with the tongue, palate and olfactory system, all of them are integrated in the brain to register as ‘tomato’. The relative amounts of, and ratios between, these components underlie consumer preference. In a recent study, consumers rated 66 different varieties, which were then dissected for their chemical constituents.^11^ Positive and negative correlations were observed between specific volatiles and liking. The results showed that sweetness was a critical contributor, mostly attributable to sugar concentrations. However, there were outliers—varieties rated as particularly sweet despite having relatively low sugar content. For example, the heirloom variety ‘Matina’ was rated as being twice as sweet as ‘Yellow Jelly Bean’ even though it had 10% less sugar.^11^ ‘Matina’ had significantly higher levels of certain volatiles than ‘Yellow Jelly Bean’, creating the illusion of sweetness in the absence of sugar. These results were consistent with previous findings that fruity volatiles enhanced the perception of sweetness,^18^ and that fruits lacking specific carotenoid volatiles were described as less sweet.^19^ Together, these results indicate that humans integrate different sensory signals, termed cross-modality, to create a brain image of flavor. Possibly through learned behavior, we come to associate certain volatiles as being paired with sweetness of fruits and the two sensations amplify each other. The results help to explain why certain volatiles are so important to consumer liking.

Although many volatiles impact consumer liking, a detailed understanding of the metabolic pathways underlying their synthesis can greatly simplify the breeding challenges. That is, many of the important volatiles are metabolically linked and there should be genetic loci that correspond to rate-limiting steps in their biosynthetic pathways. Sensory improvement may best be approached by targeting those genes associated with large sets of metabolic changes. Over 50 quantitative trait loci (QTLs) contributing to the production of influential volatiles have been identified.^20–24^ Efforts now focus on prioritizing the most influential loci encoding upstream processes that affect large ranges of metabolites, such as aromatic amino-acid decarboxylases, the first committed step in the production of key phenylpropanoid volatiles like 2-phenylethanol and 2-phenylacetaldehyde.^25^ From there, natural variation can be exploited to introduce the most desirable alleles into elite germplasm. Even transgenic or gene-edited lines may provide a rapid means to produce higher-flavored tomatoes more consistently.

Part of the flavor problem comes from inadvertent selection of genes that negatively affect quality. For instance, most modern tomato cultivars contain a mutation in a gene known as uniform ripening.^26^ The mutation is in a *Golden 2-*like transcription factor that affects chlorophyll accumulation and distribution in the fruit. Wild-type tomato fruits exhibit a progressive gradient of ripening from blossom end to stem. This manifests as a dark green shoulder on the fruit, making it appear less than fully ripe. Loss-of-function results in uniform ripening, that while more attractive, contain fewer chloroplasts and lower soluble solids, meaning that the selection for fruits with uniform ripening inherently lack the genetic potential to produce the full complement of the compounds responsible for good flavor.

The control of tomato volatile production frequently coincides with developmental transitions. For instance, ripening is a hormone-mediated process that produces changes in fruit, many which affect flavors and aromas. It is no surprise that mutations in transcription factors like RIPENING INHIBITOR (RIN) and NON-RIPENING (NOR) or the HIGH PIGMENT (HP) protein, result in pleiotropic defects in ripening progression that also change suites of flavor volatiles.^27^ Tomato ripening is ethylene dependent, so defects in ethylene synthesis, sensing or signaling also affect volatile production. For instance, the *Nr* (ever ripe) mutants are ethylene insensitive due to defects in an ethylene receptor,^28^ leading to widespread differences in volatile production in maturing fruits.^27^ Ripening also coincides with patterns of DNA demethylation, regulated by a SPB-box (SQUAMOSA promoter binding protein-like) protein COLOURLESS NON-RIPENING (CNR). The *cnr* mutants present defects in a factor required for normal ripening^29^ and show large-scale differences in compounds present.^27^ All of the ripening-impaired lines exhibit low or non-detectable levels of the *TomloxC* transcript, required for the production of C5 and C6 fatty acid-derived aldehydes and alcohols that contribute to volatile synthesis.^30^ Importantly, *rin* mutants are widely used in commercial practice to slow-down fruit ripening and extending shelf life despite their potential impacts on flavor quality.

Another area of great potential is understanding the role of enzymes that modify flavor volatiles. For instance, substantial quantities of many volatiles exist in the ripening fruit as non-volatile glycosides, effectively negating their effects on flavor.^31^ However, there are ~120 glycosyltransferases in tomato. Each volatile can be glycosylated by multiple enzymes and each enzyme can modify multiple volatiles. At this point more basic research must be performed to harness this potential mechanism of flavor improvement.

To make matters more complex, all of the above-mentioned factors are also influenced by tomato production and post-harvest handling. Tomato flavor is affected by growing conditions, time of ripening and developmental state on harvesting,^32^ degreening procedures,^33^ storage temperature^34^ and post-harvest handling.^35^ Almost nothing is known about optimizing genetics for environment to achieve maximum flavor potential.

Future breeding directions in tomato for flavor will attempt to target some of the main pathways that influence sets of important volatiles. Consumer-based testing is an important first step in prioritization of QTLs and genes, and new gene-editing approaches likely will have profound effects in designing the next generation of highly flavored tomatoes.

## Strawberry

Breeding gains in fragile strawberry fruits have allowed seasonal production in coastal climates to reach consumers across broad geographical regions. It is common to find California strawberries in Florida during the summer months in the USA, and Spanish strawberries across Europe. But like tomato, these production and distribution gains typically came at the expense of flavors and aromas. Although breeding for improved sensory traits is always a challenge, strawberry presents an additional barrier—a complicated octoploid genome that makes simple genetic relationships difficult to decipher, and adds a formidable challenge to pyramiding favorable genes or alleles into a single superior variety.

But the same genome also represents opportunity. The room for improvement is benchmarked by the explosion of fruity and floral aromas present in wild diploid strawberry species like *Fragaria vesca* and other non-octoploid fruits.^36,37^ These small, soft berries present a savory bouquet unmatched by commercial varieties. Even the hexaploid strawberry (*Fragaria moschata*) varieties present musky tones that are not apparent in octoploid cultivars, and demonstrate the breadth of strawberry’s genetic potential for improved sensory quality.

But is that potential even present in the genomes of commercial strawberries? One known limitation is that commercial varieties arise from a narrow core of foundational genotypes. It has been estimated that the entire commercial strawberry germplasm arises from fifty-some foundational lines^38^ and seventeen cytoplasms.^39^ The extensive breeding within this narrow genetic set means that the potential for exceptional flavors may be lost.

However, significant numbers of wild, interfertile octoploid strawberry species have been identified and show great variation for growth habits, stature and fruit types.^40^ These wild accessions have limited commercial value, other than to potentially serve as genetic repositories for the chemistries that may shape the future of strawberry flavors. Most of the work in this area has centered around *Fragaria chiloensis*, a wild octoploid strawberry species that grows on the western coast of Chile and North America.^41^ The white-fruited varieties of Chile are recognized for unique flavors,^42^ and have been examined for volatile content^43^ and causal genes such as alcohol acyl transferases.^44^ These species also present excellent tolerance to disease, weather extremes and pests,^45^ and may prove to be a wild repository for useful genetics in variety improvement centered around sensory and commercial quality.

Strawberry breeders have turned their attention to reclaiming these aromas that are present in the genus, but lost from commercial varieties. Consumer appreciation is related most strongly to sweetness, driven primarily by sucrose.^12^ The same report indicates that titratable acidity contributes to non-liking. The sensory experience is intensified by volatile organic compounds. While over 360 different compounds are thought to contribute to strawberry aroma,^4^ it has been estimated that a much smaller group of ~20 contributes significantly to sensory quality.^46^ Consumer tests have narrowed a select set of compounds that tend to associate with liking.^46–48^ The consensus shows appreciation for butanoic acid (plus its ethyl and methyl esters), hexanoic acid (methyl and ethyl esters), linalool and furaneol (4-hydroxy-2,5-dimethyl-3-furanone). Not surprisingly, these are compounds that impart a fruity, floral or ‘sweet’ sensation to the subject. One novel breeding approach skipped molecular markers and devised a ‘chemometric’ rubric to identify high-flavor candidates by moving directly to volatile analysis.^49^ In this case, researchers simply screened massive numbers of fruits, not for genes, but for volatiles directly. Fruits fitting a pattern of products known to positively affect flavor are tagged for further analysis. This laborious practice has the advantage of not making predictions, but relying on the olfactory needle in a haystack to identify high-flavor accessions.

Major efforts have centered around fruity volatiles. Some of the first productive microarray-based gene expression assessments in plants identified transcripts associated with the production of fruity esters, namely one encoding an alcohol acyl-transferase.^50^ The candidate transcript encoded a protein that could induce volatile ester production from medium-chain alcohols, leading to the production of a series of fruity aromatic compounds that were known to have a correlation with flavor quality.^51–53^

Key volatiles in strawberry include furaneol and its methyl ether 2,5-dimethyl-4-methoxy-3(2H)-furanone. These compounds produces a rich, buttery, caramel-like aroma. It is present in high absolute amounts in strawberry, up to 55 mg kg^−1^ fresh weight, and has a low odor threshold.^54^ A ripening-induced transcript encoding a quinone oxidoreductase was cloned from commercial strawberry, and represents the last step in furaneol biosynthesis.^55^ While this compound plays a central role in flavor perception, it is metabolized by UDP-glycosyltransferases that are induced by ripening and changes HDMF (4-hydroxy-2,5-dimethyl-3(2H)-furanone) to a relatively sensorally benign β-d-glucoside.^56^ Future efforts in gene editing may permit alteration of these modification mechanisms, leading to higher levels of this important volatile. Mesifurane is a related compound that imparts similar characteristics, only with a more ‘burnt’ or ‘sherry-like’ profile,^54^ and is produced from methylation of furaneol, orchestrated by an *O*-methyl transferase.^57,58^

Another important fruity volatile in strawberries is linalool. This compound appears to be differentially produced between diploid and octoploid strawberries, occurring only in the latter.^59^ The molecular mechanism was traced back to localization of the enzyme NEROLIDOL SYNTHASE 1 (NES1) which is present in the cytosol of linalool producing varieties. In diploid strawberries the enzyme is located in the chloroplast, availing to a different substrate pool and leading to the production of nerolidol instead of linaool. The differential partitioning is based on a premature stop codon in the commercial accessions that leads to translation beginning downstream of the normal start site, producing a functional protein capable of catalysis, yet lacking a normal chloroplast-targeting leader peptide. The linalool-producing allele is present in all commercial varieties, is lacking in other levels of ploidy, yet is missing in a subset of wild octoploids.^60^

The genes contributing to eugenol synthesis have also been functionally characterized in strawberry. Eugenol and isoeugenol are synthesized in the flowers of many plants to attract pollinators, but have been identified as important volatile compounds in ripe strawberry fruits, but it is much more prevalent in the wild diploids than in cultivated accessions.^36,57,61,62^ Its synthesis is under the control of eugenol synthase (*Fa*EGS2) and controlled by a transcription factor that is orthologous to the R2R3 MYB transcription factor controlling benzenoid emissions in petunia.^63^

The compound γ-decalactone confers a strong peach essence to fruits. Two groups identified the same underlying gene, *FAD1*, using two contrasting approaches. Sanchez-Sevilla *et al.*^64^ identified a QTL in a segregating population of commercial strawberries. They then overlaid transcriptome data to identify the gene *FAD1*, a transcript specific to the individuals that produced γ-decalactone in the mapping population. A different approach was simultaneously performed by Chambers *et al.*^65^ and came to the identical conclusion. A cross was made between a strawberry non-producer and a producer, and the compound segregated as a single dominant locus in the F1 generation. A transcriptome profile and volatile profile were obtained from individual fruits from each plant in the progeny. The transcriptomes were grouped computationally based on presence or absence of γ-decalactone, and a single transcript showed a presence/absence relationship that fit invariably with the compound’s detection. The corresponding gene was a fatty-acid desaturase, termed *FAD1*.

These analyses have shown that the volatile-rich background of strawberry can be focused down to a relatively small slate of compounds, somewhere between five and two dozen,^36,46^ that contribute to its core flavor and aromas. Most have been prioritized and narrowed down to genes associated with their synthesis and/or inactivation, and molecular markers have been developed or are being explored. Coupled to new gene-editing approaches and the implementation of genomics-level breeding tools, strawberry flavor stands to improve from its current state, where production traits have been valued over sensory characteristics.

## Conclusions

Tomato and strawberry present two contrasting yet complementary systems. The findings from tomato show that most genes identified in flavor presentation control suites of genes and metabolites. Strawberry, on the other hand, shows contributions from specific genes with roles in discrete biosynthetic pathways. Both suffer from years of efficient breeding for production characteristics that have resulted in wide availability of fruits and vegetables but there is plenty of room to improve flavors and aromas. New approaches can be thought of as ‘consumer-assisted selection’, allowing the consumer with his/her complicated sensory preferences to prioritize the next steps in the breeding process. Coupling the targets defined by consumer tests to today’s powerful metabolomic and genomic technologies, plus the potential for genome editing, suggests that creating the next wave of highly flavored fruits and vegetables can be streamlined compared with yesterday’s processes of traditional breeding. The tomatoes and strawberries of the future will carry the best of modern production qualities and the sensory experience of a garden-grown heirloom variety.

## Figures and Tables

**Figure 1:**
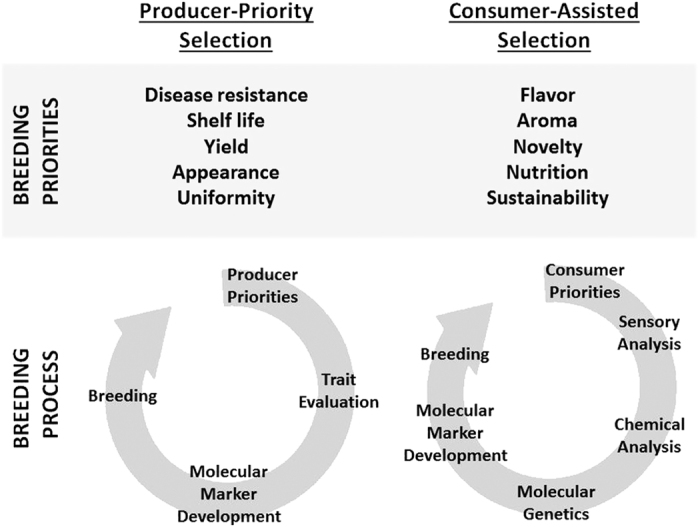
The changing priorities in variety selection. Traditionally plant genetic improvement was driven by the demands of the end user—the growers. Breeding and selection efforts prioritized production and post-harvest characteristics. Today the process is shifting to a model where the consumer is the end user, and their demands are quite different. Future efforts in breeding and selection will use this ‘Consumer-Assisted’ strategy, allowing consumers to define immediate improvement priorities.

**Figure 2:**
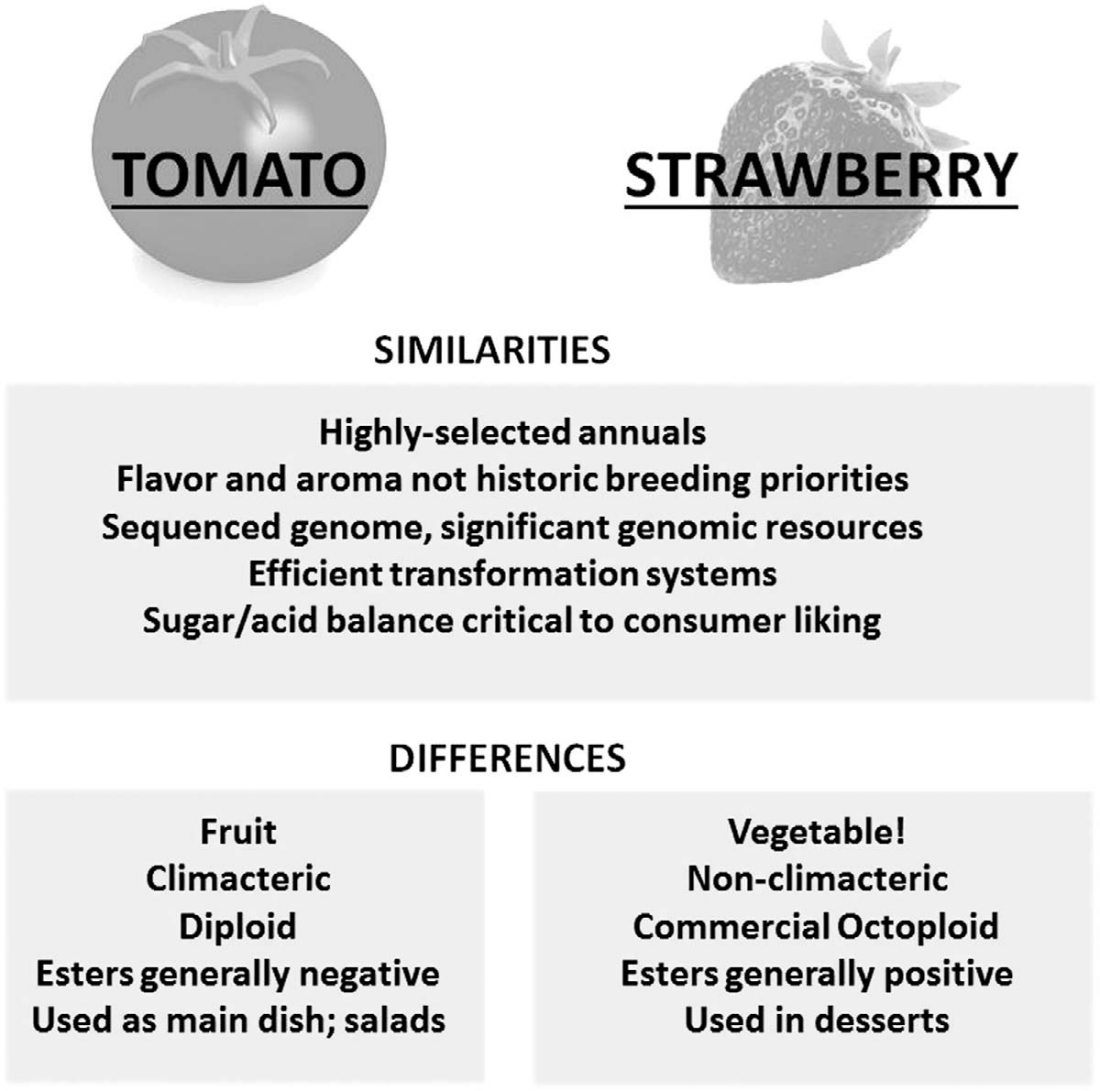
Comparison and contrast of the tomato and strawberry systems. Tomato and strawberry biology is well understood, and new genomics tools make gene discovery and validation possible. These platforms allow understanding of the molecular basis of flavors and aromas that consumers find desirable. However, both ‘fruits’ have unique aspects based on their botanical origin, development and chemistry, making them complementary models for implementing consumer-assisted selection.

## References

[bib1] Ford H . My Life and Work. Kessinger Publishing: New York, NY, 2003, 296.

[bib2] Bruhn CM , Feldman N , Garlitz C , Harwood J , Ivans E , Marshall M et al. Consumer perceptions of quality: apricots, cantaloupes, peaches, pears, strawberries, and tomatoes. J Food Qual 1991; 14: 187–195.

[bib3] Mirsky S . Bacon, lettuce and tasteless. Sci Am 2013; 308: 84–84.23469438

[bib4] Latrasse A. Fruits. In: Maarse H (ed). Volatile Compounds in Foods and Beverages. Marcel-Decker: New York, NY. 1991, 329–387.

[bib5] Baldwin EA , Goodner K , Plotto A . Interaction of volatiles, sugars, and acids on perception of tomato aroma and flavor descriptors. J Food Sci 2008; 73: S294–S307. 19241574 10.1111/j.1750-3841.2008.00825.x

[bib6] Petro‐Turza M . Flavor of tomato and tomato products. Food Rev Int 1986; 2: 309–351.

[bib7] Hasin-Brumshtein Y , Lancet D , Olender T . Human olfaction: from genomic variation to phenotypic diversity. Trends Genet 2009; 25: 178–184. 19303166 10.1016/j.tig.2009.02.002

[bib8] Goff SA , Klee HJ . Plant volatile compounds: sensory cues for health and nutritional value? Science 2006; 311: 815–819. 16469919 10.1126/science.1112614

[bib9] Scott JW . A breeder's perspective on the use of molecular techniques for improving fruit quality. HortScience 2002; 37: 464–467.

[bib10] Linda MB , Harry JK . Better fruits and vegetables through sensory analysis. Current Biology 2013; 23: R374–R378. 23660360 10.1016/j.cub.2013.03.038

[bib11] Tieman D , Bliss P , McIntyre LM , Blandon-Ubeda A , Bies D , Odabasi AZ et al. The chemical interactions underlying tomato flavor preferences. Curr Biol 2012; 22: 1035–1039. 22633806 10.1016/j.cub.2012.04.016

[bib12] Schwieterman M , Colquhoun TA , Jaworski EA , Bartoshuk LM , Gilbert JL , Tieman DM et al. Strawberry flavor: diverse chemical recipies, a seasonal influence and their effect on sensory perception. PLoS One 2014; 9: e88446. 24523895 10.1371/journal.pone.0088446 PMC3921181

[bib13] Harel-Beja R , Tzuri G , Portnoy V , Lotan-Pompan M , Lev S , Cohen S et al. A genetic map of melon highly enriched with fruit quality QTLs and EST markers, including sugar and carotenoid metabolism genes. Theor Appl Genet 2010; 121: 511–533. 20401460 10.1007/s00122-010-1327-4

[bib14] Senapati SK . A review on research progress on *in vitro* regeneration and transformation of tomato. Annu Res Rev Biol 2016; 9: 1–9.

[bib15] Folta KM , Dhingra A . Transformation of strawberry: the basis for translational genomics in Rosaceae. In Vitro Cell Dev Biol Plant 2006; 42: 482–490.

[bib16] Shulaev V , Sargent DJ , Crowhurst RN , Mockler TC , Folkerts O , Delcher AL et al. The genome of woodland strawberry (*Fragaria vesca*). Nat Genet 2011; 43: 109–116. 21186353 10.1038/ng.740 PMC3326587

[bib17] Tomato Genome Consortium. The tomato genome sequence provides insights into fleshy fruit evolution. Nature 2012; 485: 635–641. 22660326 10.1038/nature11119 PMC3378239

[bib18] Baldwin EA , Scott JW , Einstein MA , Malundo TM , Carr BT , Shewfelt RL et al. Relationship between sensory and instrumental analysis for tomato flavor. J Am Soc Hortic Sc 1998; 123: 906–915.

[bib19] Vogel JT , Tieman DM , Sims CA , Odabasi AZ , Clark DG , Klee HJ . Carotenoid content impacts flavor acceptability in tomato (*Solanum lycopersicum*). J Sci Food Agric 2010; 90: 2233–2240. 20661902 10.1002/jsfa.4076

[bib20] Tieman DM , Zeigler M , Schmelz EA , Taylor MG , Bliss P , Kirst M et al. Identification of loci affecting flavour volatile emissions in tomato fruits. J Exp Bot 2006; 57: 887–896. 16473892 10.1093/jxb/erj074

[bib21] Causse M , Saliba-Colombani V , Lecomte L , Duffé P , Rousselle P , Buret M . QTL analysis of fruit quality in fresh market tomato: a few chromosome regions control the variation of sensory and instrumental traits. J Exp Bot 2002; 53: 2089–2098. 12324532 10.1093/jxb/erf058

[bib22] Mathieu S , Cin VD , Fei Z , Li H , Bliss P , Taylor MG et al. Flavour compounds in tomato fruits: identification of loci and potential pathways affecting volatile composition. J Exp Bot 2009; 60: 325–337. 19088332 10.1093/jxb/ern294 PMC3071775

[bib23] Saliba-Colombani V , Causse M , Langlois D , Philouze J , Buret M . Genetic analysis of organoleptic quality in fresh market tomato. 1. Mapping QTLs for physical and chemical traits. Theor Appl Genet 2001; 102: 259–272.

[bib24] Lecomte L , Duffé P , Buret M , Servin B , Causse M . Marker-assisted introgression of five QTLs controlling fruit quality traits into three tomato lines revealed interactions between QTLs and genetic backgrounds. Theor Appl Genet 2004; 109: 658–668. 15112037 10.1007/s00122-004-1674-0

[bib25] Tieman D , Taylor M , Schauer N , Fernie AR , Hanson AD , Klee HJ . Tomato aromatic amino acid decarboxylases participate in synthesis of the flavor volatiles 2-phenylethanol and 2-phenylacetaldehyde. Proc Natl Acad Sci USA 2006; 103: 8287–8292. 16698923 10.1073/pnas.0602469103 PMC1472464

[bib26] Powell ALT , Nguyen CV , Hill T , Cheng KL , Figueroa-Balderas R , Aktas H et al. Uniform ripening Encodes a Golden 2-like transcription factor regulating tomato fruit chloroplast development. Science 2012; 336: 1711–1715. 22745430 10.1126/science.1222218

[bib27] Kovács K , Fray RG , Tikunov Y , Graham N , Bradley G , Seymour GB et al. Effect of tomato pleiotropic ripening mutations on flavour volatile biosynthesis. Phytochemistry 2009; 70: 1003–1008. 19539963 10.1016/j.phytochem.2009.05.014

[bib28] Wilkinson JQ , Lanahan MB , Yen H-C , Giovannoni JJ , Klee HJ . An ethylene-inducible component of signal transduction encoded by never-ripe. Science 1995; 270: 1807–1809. 8525371 10.1126/science.270.5243.1807

[bib29] Manning K , Tör M , Poole M , Hong Y , Thompson AJ , King GJ et al. A naturally occurring epigenetic mutation in a gene encoding an SBP-box transcription factor inhibits tomato fruit ripening. Nat Genet 2006; 38: 948–952. 16832354 10.1038/ng1841

[bib30] Chen G , Hackett R , Walker D , Taylor A , Lin Z , Grierson D . Identification of a specific isoform of tomato lipoxygenase (TomloxC) involved in the generation of fatty acid-derived flavor compounds. Plant Physiol 2004; 136: 2641–2651. 15347800 10.1104/pp.104.041608 PMC523329

[bib31] Tikunov YM , de Vos RCH , González Paramás AM , Hall RD , Bovy AG . A role for differential glycoconjugation in the emission of phenylpropanoid volatiles from tomato fruit discovered using a metabolic data fusion approach. Plant Physiol 2010; 152: 55–70. 19889876 10.1104/pp.109.146670 PMC2799346

[bib32] Dorais M , Papadopoulos AP , Gosselin A . Greenhouse Tomato Fruit Quality. In: Jules Janick (ed.). Horticultural Reviews. Oxford: John Wiley & Sons, Inc., 2000; 239–319.

[bib33] Baldwin E , Plotto A , Narciso J , Bai J . Effect of 1-methylcyclopropene on tomato flavour components, shelf life and decay as influenced by harvest maturity and storage temperature. J Sci Food Agric 2011; 91: 969–980. 21337573 10.1002/jsfa.4281

[bib34] Maul F , Sargent SA , Sims CA , Baldwin EA , Balaban MO , Huber DJ . Tomato flavor and aroma quality as affected by storage temperature. J Food Sci 2000; 65: 1228–1237.

[bib35] Zushi K , Matsuzoe N . Postharvest changes in sugar, organic acid, glutamic acid and antioxidant contents in tomato fruit grown under salinity stress. Environ Control Biol 2006; 44: 111–117.

[bib36] Ulrich D , Komes D , Olbricht K , Hoberg E . Diversity of aroma patterns in wild and cultivated Fragaria accessions. Genet Resour Crop Evol 2007; 54: 1185–1196.

[bib37] Ulrich D , Olbricht K . Diversity of metabolite patterns and sensory characters in wild and cultivated strawberries. J Berry Res 2014; 4: 11–17.

[bib38] Sjulin TM , Dale A . Genetic diversity of North-American strawberry cultivars. J Am Soc Hortic Sci 1987; 112: 375–385.

[bib39] Dale A , Sjulin TM . Few cytoplasms contribute to North-American strawberry cultivars. Hortscience 1990; 25: 1341–1342.

[bib40] Hancock JF , Callow PW , Dale A , Luby J , Finn CE , Hokanson SC et al. From the andes to the rockies: native strawberry collection and utilization. Hortscience 2001; 36: 221–225.

[bib41] Carrasco B , Garcés M , Rojas P , Saud G , Herrera R , Retamales JB et al. The Chilean strawberry [*Fragaria chiloensis* (L.) Duch.]: genetic diversity and structure. J Am Soc Hortic Sci 2007; 132: 501–506.

[bib42] Adasme C , Spiller A , Diaz J . Determinación de Preferencias del Consumidor de la Región Metropolitana hacia la frutilla blanca (*Fragaria chiloensis*). Un Análisis Conjunto y una Prueba Sensorial. Economía Agraria 2006; 10: 1–10.

[bib43] Prat L , Espinoza MI , Agosin E , Silva H . Identification of volatile compounds associated with the aroma of white strawberries (*Fragaria chiloensis*). J Sci Food Agric 2014; 94: 752–759. 24115051 10.1002/jsfa.6412

[bib44] González M , Gaete-Eastman C , Valdenegro M , Figueroa CR , Fuentes L , Herrera R et al. Aroma development during ripening of *Fragaria chiloensis* fruit and participation of an alcohol acyltransferase (FcAAT1) gene. J Agric Food Chem 2009; 57: 9123–9132. 19731914 10.1021/jf901693j

[bib45] Retamales JB , Caligari PDS , Carrasco B , Saud G . Current status of the Chilean native strawberry and the research needs to convert the species into a commercial crop. Hortscience 2005; 40: 1633–1634.

[bib46] Schieberle P , Hofmann T . Evaluation of the character impact odorants in fresh strawberry juice by quantitative measurements and sensory studies on model mixtures. J Agric Food Chem 1997; 45: 227–232.

[bib47] Ulrich D , Hoberg E , Rapp A , Kecke S . Analysis of strawberry flavour - discrimination of aroma types by quantification of volatile compounds. Z Lebensm Unters Forsch A 1997; 205: 218–223.

[bib48] Jouquand C , Chandler C , Plotto A , Goodner K . A sensory and chemical analysis of fresh strawberries over harvest dates and seasons reveals factors that affect eating quality. J Am Soc Hortic Sci 2008; 133: 859–867.

[bib49] Vandendriessche T , Geerts P , Membrebe BN , Keulemans J , Nicolaï BM , Hertog ML . Journeys through aroma space: a novel approach towards the selection of aroma-enriched strawberry cultivars in breeding programmes. Plant Breed 2013; 132: 217–223.

[bib50] Aharoni A , Keizer LC , Bouwmeester HJ , Sun Z , Alvarez-Huerta M , Verhoeven HA et al. Identification of the SAAT gene involved in strawberry flavor biogenesis by use of DNA microarrays. Plant Cell 2000; 12: 647–662. 10810141 10.1105/tpc.12.5.647 PMC139918

[bib51] Pérez AG , Sanz C , Olías R , Ríos JJ , Olías JM . Evolution of strawberry alcohol acyltransferase activity during fruit development and storage. J Agric Food Chem 1996; 44: 3286–3290.

[bib52] Carbone F , Mourgues F , Biasioli F , Gasperi F , Märk TD , Rosati C et al. Development of molecular and biochemical tools to investigate fruit quality traits in strawberry elite genotypes. Mol Breed 2006; 18: 127–142.

[bib53] Cumplido-Laso G , Medina-Puche L , Moyano E , Hoffmann T , Sinz Q , Ring L et al. The fruit ripening-related gene FaAAT2 encodes an acyl transferase involved in strawberry aroma biogenesis. J Exp Bot 2012; 63: 4275–4290. 22563120 10.1093/jxb/ers120

[bib54] Larsen M , Poll L . Odour thresholds of some important aroma compounds in strawberries. Z Lebensum Unters Forsch 1992; 195: 120–123.

[bib55] Raab T , López-Ráez JA , Klein D , Caballero JL , Moyano E , Schwab W et al. FaQR, required for the biosynthesis of the strawberry flavor compound 4-hydroxy-2,5-dimethyl-3(2H)-furanone, encodes an enone oxidoreductase. Plant Cell 2006; 18: 1023–1037. 16517758 10.1105/tpc.105.039784 PMC1425863

[bib56] Song C , Hong X , Zhao S , Liu J , Schulenburg K , Huang FC et al. Glucosylation of 4-hydroxy-2,5-dimethyl-3(2H)-furanone, the key strawberry flavor compound in strawberry fruit. Plant Physiol 2016; 171: 139–151. 26993618 10.1104/pp.16.00226 PMC4854714

[bib57] Zorrilla-Fontanesi Y , Rambla JL , Cabeza A , Medina JJ , Sánchez-Sevilla JF , Valpuesta V . Genetic analysis of strawberry fruit aroma and identification of *O*-methyltransferase FaOMT as the locus controlling natural variation in mesifurane content. Plant Physiol 2012; 159: 851–870. 22474217 10.1104/pp.111.188318 PMC3375946

[bib58] Lunkenbein S , Salentijn EM , Coiner HA , Boone MJ , Krens FA , Schwab W . Up- and down-regulation of Fragariaxananassa *O*-methyltransferase: impacts on furanone and phenylpropanoid metabolism. J Exp Bot 2006; 57: 2445–2453. 16798852 10.1093/jxb/erl008

[bib59] Aharoni A , Giri AP , Verstappen FW , Bertea CM , Sevenier R , Sun Z et al. Gain and loss of fruit flavor compounds produced by wild and cultivated strawberry species. Plant Cell 2004; 16: 3110–3131. 15522848 10.1105/tpc.104.023895 PMC527202

[bib60] Chambers A , Whitaker VM , Gibbs B , Plotto A , Folta KM . Detection of the linalool-producing NES1 variant across diverse strawberry (Fragaria spp.) accessions. Plant Breed 2012; 131: 437–443.

[bib61] Pyysalo T , Honkanen E , Hirvi T . Volatiles of wild strawberries, Fragaria-Vesca L, compared to those of cultivated berries, Fragaria X Ananassa Cv Senga Sengana. J Agric Food Chem 1979; 27: 19–22.

[bib62] Zorrilla-Fontanesi Y , Cabeza A , Domínguez P , Medina JJ , Valpuesta V , Monfort A et al. Quantitative trait loci and underlying candidate genes controlling agronomical and fruit quality traits in octoploid strawberry (Fragaria×ananassa). Theor Appl Genet 2011; 123: 755–778. 21667037 10.1007/s00122-011-1624-6

[bib63] Verdonk JC , Haring MA , van Tunen AJ , Schuurink RC . ODORANT1 regulates fragrance biosynthesis in petunia flowers. Plant Cell 2005; 17: 1612–1624. 15805488 10.1105/tpc.104.028837 PMC1091778

[bib64] Sánchez-Sevilla JF , Cruz-Rus E , Valpuesta V , Botella MA , Amaya I . Deciphering gamma-decalactone biosynthesis in strawberry fruit using a combination of genetic mapping, RNA-Seq and eQTL analyses. BMC Genomics 2014; 15: 1–15. 24742100 10.1186/1471-2164-15-218 PMC4023230

[bib65] Chambers A , Pillet J , Plotto A , Bai J , Whitaker VM , Folta KM . Identification of a strawberry flavor gene candidate using an integrated genetic-genomic-analytical chemistry approach. BMC Genomics 2014; 15: 217. 24742080 10.1186/1471-2164-15-217 PMC4023330

